# Difference in single-leaf and whole-plant photosynthetic response to light under steady and non-steady states in *Arabidopsis thaliana*

**DOI:** 10.3389/fpls.2025.1532522

**Published:** 2025-02-18

**Authors:** Kazuma Sakoda, Atsushi Sakurai, Sousuke Imamura

**Affiliations:** Space Environment and Energy Laboratories, NTT Corporation, Tokyo, Japan

**Keywords:** Arabidopsis, photosynthesis, gas exchange, fluctuating light, stomatal conductance, light response, apparent quantum yield

## Abstract

Although photosynthetic response to light has been extensively studied at the single-leaf level, little is known about the response at the whole-plant level. The present study aims to reveal the differences in the photosynthetic response to light under steady and non-steady states between the single leaf and whole plant in *Arabidopsis thaliana* and to investigate the mechanisms underlying these differences with respect to leaf aging. First, we developed an open system for gas exchange measurement of the whole plant of Arabidopsis. It enabled the photosynthetic response to dynamic environmental changes to be directly compared between the single leaf and whole plant. The photosynthetic response to the fluctuating light did not differ significantly between the single leaf and whole plant. This result is partly confirmed by the fact that the leaves at different ages showed no difference in the photosynthetic induction after a step change in light. On the other hand, light response analysis for steady-state photosynthesis showed a higher apparent quantum yield in the whole plant than in the single leaf. This difference might be attributed to the difference in the efficiency of light absorption and/or utilization of absorbed light among the leaves at different ages.

## 1 Introduction

Photosynthesis has been an attractive target for enhancing plant growth and yield, owing to its physiological role that determines biomass production in plants (Long et al., 2006). In field conditions, environmental factors including temperature, humidity, light intensity, and CO_2_ concentration dynamically change over short to long terms. Over a short term, environmental factors can fluctuate on a time scale from seconds to hours, which impacts photosynthetic performance and, consequently, biomass production in plants (Yamori, 2016). Thus, there have been efforts to elucidate the photosynthetic response to environmental fluctuations as well as its regulatory mechanisms in plants. This knowledge can provide promising pathways toward improving biomass production by plants in the field through a genetic engineering approach (Kromdijk et al., 2016).

Given the critical role of light as a primary driver of photosynthesis, previous studies have investigated the photosynthetic response to light intensity and quality (Ögren and Evans, 1993; Loreto et al., 2009; Terashima et al., 2009). During crop growing seasons, light intensity often exceeds 2,000 µmol photons m^-2^·s^-1^ on clear days, with fluctuations occurring within less than seconds to minutes due to the changes in solar angle, cloud cover, and self- or mutual shading by plant canopies (Tanaka et al., 2019). This motivated previous researchers to investigate the photosynthetic response to light under steady and non-steady states. Following a step increase in light, the CO_2_ assimilation rate reaches the steady state with a gradual increase, which is commonly referred to as “photosynthetic induction” (Pearcy and Seemann, 1990). It was simulated that the potential loss of daily carbon gain due to photosynthetic induction can exceed 20% in crops in fields (Taylor and Long, 2017; Tanaka et al., 2019). Importantly, faster photosynthetic induction contributed to greater carbon gain and biomass production in Arabidopsis [*Arabidopsis thaliana* (L.)] under fluctuating light conditions (Papanatsiou et al., 2019; Kimura et al., 2020; Sakoda et al., 2020). These facts emphasize the need to elucidate the photosynthetic response to light under steady and non-steady states for understanding the physiological basis of biomass production of plants in fields.

Most previous studies analyzed the photosynthetic response to light at the single-leaf level, so little is known about the response at the whole-plant level (Eyland et al., 2021). Land plants typically consist of many leaves differing in age and spatial arrangement. It has been reported that the apparent quantum yield based on the light response curve of steady-state photosynthesis and the speed of photosynthetic induction differ among leaves at different ages in tomatoes [*Solanum lycopersicum* (L.)] (Zhang et al., 2022). Moreover, photosynthetic response to the fluctuating light differs among leaves at different positions within a rice [*Oryza sativa* (L.)] canopy (Acevedo-Siaca et al., 2021). Several studies have shown that the speed of leaf photosynthetic induction differs when a single leaf or whole plant is illuminated, which evidences the systemic-regulatory mechanisms of photosynthetic induction (Guo et al., 2016; Shimadzu et al., 2019). These results suggest that the observable photosynthetic response to light can differ between the single leaf and whole plant. To bridge the gap between the knowledge at the single-leaf and whole-plant levels, the difference in photosynthetic response to light under steady and non-steady states and the underlying mechanisms need to be revealed. This has the potential to provide a novel insight into the biomass production by field-grown plants, because the biomass production depends on the combined carbon gain through photosynthesis in all leaves rather than in a single leaf (Long et al., 2006).

The objective of the present study is to reveal the photosynthetic response to light under steady and non-steady states in the whole plant and its difference from the single-leaf response in plants. To achieve this, we first developed a custom chamber for the whole plant of Arabidopsis that can be connected to an open gas-exchange measurement system. This enables the direct comparison of photosynthetic response to dynamic environmental changes between the single leaf and whole plant. The photosynthetic response to the fluctuating light was compared in the single leaf and whole plant under singular and repeatedly changing light conditions. The light response curve of steady-state photosynthesis (i.e., *A*-Q curve) was also analyzed to evaluate the apparent quantum yields. Furthermore, we investigated the photosynthetic induction and *A*-Q curve in the single leaf through the aging process to discuss the mechanisms underlying the difference in the photosynthetic response to light between the single leaf and whole plant.

## 2 Materials and methods

### 2.1 Plant materials and cultivation

The present study used Columbia-0 (CS60000) of Arabidopsis. In the growth chamber, Arabidopsis plants were grown in equivalent mixtures of vermiculite and nutrient soil (Metro-Mix; Sun Gro Horticulture, Agawam, MA, USA) at an air humidity of 70% and a photosynthetic photon flux density (PPFD) of 100 µmol photons m^-2^ s^-1^. The day/night length was 10/14h with a constant air temperature of 22°C. Throughout the growth period, a plant distribution in the growth chamber was randomly arranged every 3-4 days to minimize the spacing effects. The plants at 33-66 days after the sowing were used for the gas exchange measurements before they began bolting.

### 2.2 Chamber specifications for gas exchange measurement at a single leaf and whole plant in Arabidopsis

**Figure 1:**
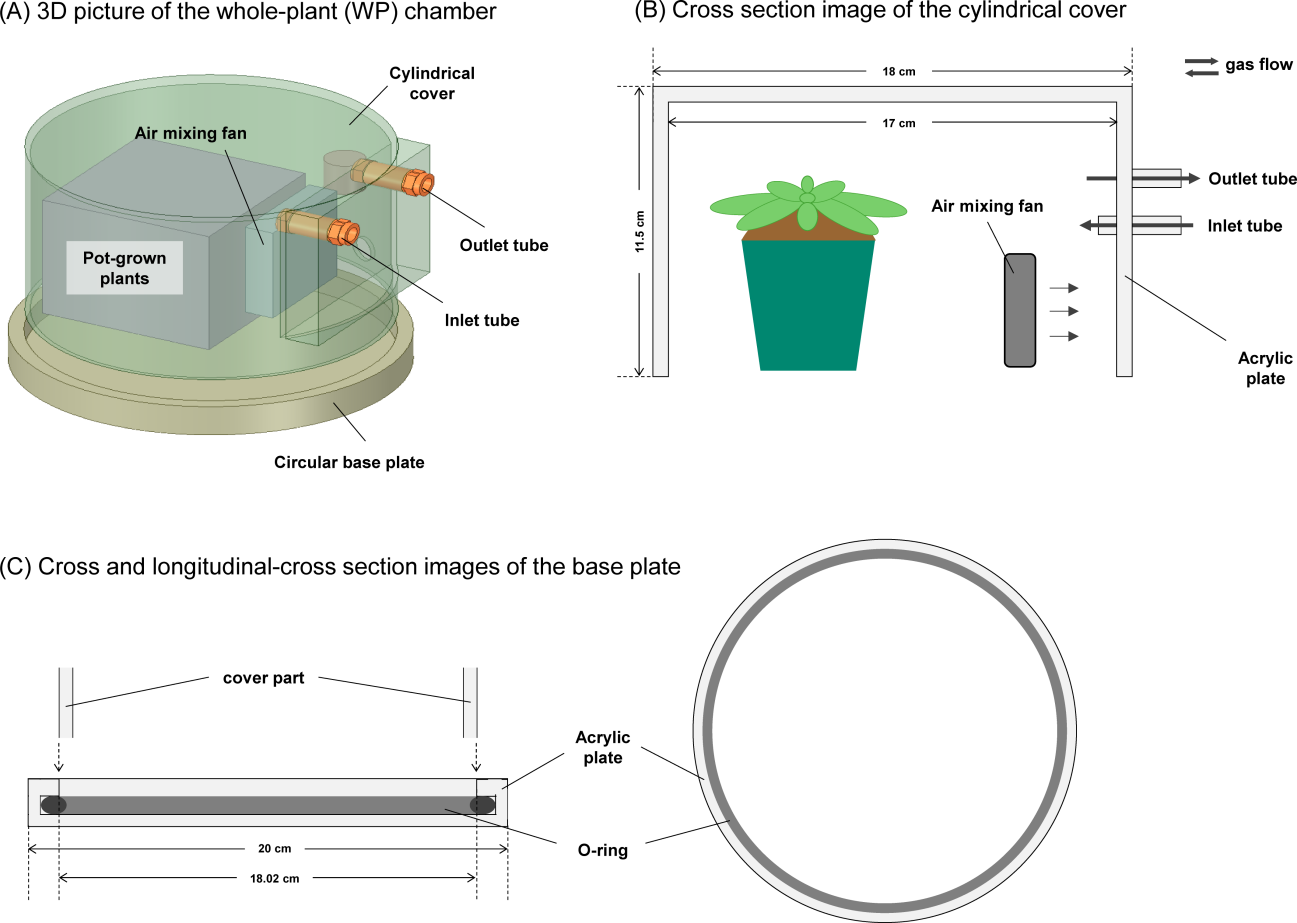
The design of the whole plant chamber for gas exchange measurements. **(A)** 3D picture of the chamber for gas exchange measurements with the whole plant of Arabidopsis. The chamber consists of two parts: **(B)** a cylindrical cover with an opened bottom and **(C)** a circular base plate. The cylindrical cover is equipped with an air-mixing fan where the rotation speed can be controlled depending on the applied voltage. In addition, it has two holes for an air inlet and outlet connected to an open gas exchange measurement system via a tube. The base plate is equipped with an O-ring to prevent gas leaks.

For gas exchange measurement of a single leaf, we used the clear-top chamber (leaf chamber) with a volume of 102 cm^3^ connectable to an open gas-exchange measurement system, LI-6800 (*LI-COR*, Lincoln, NE, USA) (**Supplementary Figure S1A**). The top of the leaf chamber is covered by Propafilm with a high transparency. Moreover, we constructed the specialized chamber (whole plant (WP) chamber) to measure gas exchange in a whole plant of Arabidopsis (**Figure 1**). The chamber with a volume of 2495.5 cm^3^ is made of acrylic and consists of two parts: (1) a cylindrical cover with an opened bottom and (2) a circular base plate. A thickness of a cylindrical cover is 0.5 cm. The base plate is equipped with an O-ring to prevent gas leaks. The cylindrical cover is equipped with an air-mixing fan where the rotation speed can be controlled depending on the applied voltage as shown in **Supplementary Figure S2**. In addition, it has two holes for an air inlet and outlet connected to LI-6800 via a silicone tube.

We measured spectral properties of a direct light from the light-emitting diodes (LEDs) in the growth chamber and the light transmitted through Propafilm and acryl used for the leaf and WP chambers, respectively, by using a spectroradiometer (USR45, Ushio Inc., Tokyo, Japan) (**Supplementary Figure S1**).

### 2.3 Evaluation of the effects of fan speed and flow rate on photosynthetic response to light

The leaf and WP chambers largely differ in terms of air-mixing speed and volume. The wind speed was previously demonstrated to affect the leaf boundary layer conductance and, subsequently, CO_2_ assimilation rate (Kitaya et al., 2003). The difference in an air-mixing speed controlled by the fan results in the difference in wind speed, which can affect the evaluation of photosynthetic response to light. Furthermore, the volume difference between two chambers leads to differential gas-replacement speeds at an equivalent flow rate. This potentially induces the difference in the detection sensitivity to [CO_2_] changes due to photosynthesis and, in turn, can affect the evaluation of photosynthetic response. Considering these facts, we first examined the effects of the differences in a fan speed and flow rate on the photosynthetic response at a single leaf level. The CO_2_ assimilation rate per unit leaf area (*A*) was measured using the leaf chamber at 10-sec intervals after a step increase in light from a PPFD of 100 for 5 min to 500 µmol photons m^-2^ s^-1^ for 15 min at a flow rate of 120, 200, or 300 µmol s^-1^ and a fan speed of 8000 rpm, or a fan speed of 3000, 5000, or 8000 rpm and a flow rate of 300 µmol s^-1^, with [CO_2_] of 400 µmol mol^-1^, relative humidity (RH) of 55-75%, and air temperature of 26°C. Moreover, *A* was measured using the WP chamber at wind speeds of 0.35, 0.59, or 0.75 m s^-1^, respectively, with a flow rate of 1600 µmol s^-1^, under the same conditions of light, [CO_2_], RH, and air temperature in the single leaf measurement. Arabidopsis plants were illuminated under a PPFD of 100 µmol photons m^-2^ s^-1^ for more than 60 min before the measurements.

### 2.4 Comparison of gas-replacement speeds between the leaf and whole-plant chambers

To compare the rapidity of photosynthetic response to the fluctuating light using leaf and WP chambers, the flow rates at which the gas-replacement speed becomes equivalent between two chambers need to be clarified. For this objective, the change in [CO_2_] of the sample gas ([CO_2_S]) was measured at 10-sec intervals for 10 minutes when targeted [CO_2_] of the reference gas ([CO_2_R]) was changed between 300 and 400 μmol mol^-1^ at flow rates of 100, 125, 150, 175, and 200 μmol s^-1^ for the leaf chamber and 800, 1000, 1250, 1500 and 1600 μmol s^-1^ for the WP chamber. For the measurements conducted using the WP chamber, a dummy object with the same volume as a soil-filled pot (≒284 cm^3^) was placed in the chamber. Subsequently, we calculated the time when [CO_2_S] reached 350 μmol mol^-1^, defined as *t*_50_CO2S_. For each chamber, *t*_50_CO2S_ was calculated when [CO_2_R] changed from 400 to 300 μmol mol^-1^ or 300 to 400 μmol mol^-1^, and it was plotted against the flow rates. The functions representing *t*_50_CO2S_ as a variable of the flow rate were derived through curve fitting for each chamber. Employing these curve-fitted functions, we calculated the flow rate at which *t*_50_CO2S_ is equal between two chambers. Curve fitting was performed by using the curve fitting tool in the SciPy optimize module of Python (Python Software Foundation, Wilmington, DE, USA).

### 2.5 Evaluation of photosynthetic response to light under the steady and non-steady states at single-leaf and whole-plant levels

Gas exchange measurements were performed inside the growth chamber maintained at a RH of 50%, and air temperature of 26℃ for the single leaf measurement and 22℃ for the whole plant measurement (**Supplementary Figure S1**). In addition, the air conditions blown into leaf and WP chambers were targeted at [CO_2_] of 400 μmol mol^-1^ and a temperature of 26℃. The flow rates were 122 and 1600 μmol s^-1^ for the leaf chamber and WP chamber, respectively, where the gas-replacement speed becomes equivalent between two chambers. The fan speed was 3000 rpm for the leaf chamber, and the wind speed was 0.35 m s^-1^ for the WP chamber. For the leaf chamber measurements, the largest and undamaged leaf was selected from each plant. Singular and repeatedly changing light conditions were generated by LED lights placed at the top and side of the growth chamber. (**Supplementary Figure S1**). The singular changing light condition comprised darkness for 5 min and a PPFD of 500 μmol photons m^-2^ s^-1^ for 80 min (**Supplementary Figure S3A**). The repeatedly changing light condition comprised darkness for 5 min at the beginning, followed by 10 cycles of a PPFD of 60 and 500 μmol·photons·m^-2^·s^-1^ for 3 min (**Supplementary Figure S3B**). In addition, the repeatedly changing light condition was followed by a PPFD of 500 μmol·photons·m^-2^·s^-1^ for 20 min to achieve the full *A* induction. The *A* was recorded at 10-sec intervals under two light conditions. After *A* reached a steady state under a PPFD of 500 μmol·photons·m^-2^·s^-1^, *A* was measured under PPFD changed in the order of 150, 125, 100, 75, 60, and 0 μmol·photons·m^-2^·s^-1^ at 3 min intervals to analyze the light response curve of a steady-state *A* (i.e., *A*-Q curve) at [CO_2_R] of 400 and 1000 μmol·mol^-1^. Before all the measurements, Arabidopsis plants were left overnight to adapt them to darkness. To avoid the effect of systemic regulation on the *A* measurement at the single leaf, the whole part not only the measured leaf was illuminated under a PPFD of 500 μmol·photons·m^-2^·s^-1^.

As for the WP chamber measurements, the observed CO_2_ assimilation rate includes the CO_2_ emissions from the soil and roots. To eliminate the effect of these CO_2_ emissions, gas exchange measurements with the whole plant were followed by the measurement with the soil and roots after cutting off the above-ground parts of plants. The CO_2_ assimilation rate for a whole plant was calculated by subtracting the values obtained from the soil and root measurements from those obtained from the whole-plant measurements (**Supplementary Figure S4**). The *A* was calculated by dividing the CO_2_ assimilation rate for whole plant by the total leaf area. Here, we first examined the relationship between the projected leaf area (PLA) and actual leaf area (ALA) measured by the destruction of the plant to separate each leaf. The PLA and ALA (cm^2^) were measured by using imaging analysis software, ImageJ (NIH, Bethesda, MD, USA). The relationship between PLA and ALA clearly fits the linear regression described as ALA = 1.1264 x PLA -1.5221 (**Supplementary Figure S5A**). Using this equation, we estimated ALA from the PLA and then calculated *A* on the basis of ALA.

To examine the leaf-aging effect on photosynthetic response to light at the single leaf level, we conducted gas exchange measurements under the singular changing light condition and *A*-Q curve analysis on the same leaf at seven-day intervals over a period of four weeks (**Supplementary Figure S6**). In addition to *A*, the stomatal conductance (*g*_s_) and intercellular CO_2_ concentration (*C*_i_) was recorded during the measurements. To evaluate the induction of *A* corrected for stomatal limitation, *A* assuming *C*_i_ = 300 μmol mol ^-1^ (*A* ^*^) was calculated during the photosynthetic induction as described in Soleh et al., 2016. The assumed *C*_i_ value can be set within the dynamic range of *C*_i_ during the measurement. The present study assumed *C*_i_ = 300 μmol mol ^-1^ because *C*_i_ mostly ranged within 200 to 400 μmol mol ^-1^ during the measurements.

### 2.6 Definition of the parameters related to photosynthetic response to light

To evaluate induction kinetics of *A*, *A* ^*^, and *g*_s_, we calculated the normalized values of each parameter (*A*_ind_, *A* ^*ind^, and *g*_sind_) defined as the following equations as described by Sakoda et al. (2020):


$$X_{\text{ind}}\text{ = }\frac{X_{\text{t}}{\text{ - }X}_{\text{min}}}{X_{\text{max}}{\;}_{\;}{\text{- }X}_{\text{min}}}$$

where *X*_min_ represents the steady-state values under darkness or a PPFD of 100 µmol photons m^-2^ s^-1^ prior to the high-light illumination, *X*_max_ represents the maximum values under a PPFD of 500 μmol·photons·m^-2^ s^-1^, and *X_t_* represents values at a given time after a step change in light intensity. We evaluated the time when *X*_ind_ reached the closest values of 50%, 60%, 70%, 80%, and 90% to the maximum values (*t*_50_ - *t*_90_) under the singular and repeatedly changing light conditions. For the repeatedly changing light condition, *A*_ind_ was compared between the single leaf and whole plant at 1, 2 and 3 min after the light transient from low to high light in 1-10 cycles. The cumulative CO_2_ assimilation (*CCA*) was calculated by summing *A* under the repeatedly changing light condition. In the *A*-Q curve analysis, we calculated the initial slope as the apparent quantum yield between *A* and light intensities under a PPFD of 60, 75, 100 µmol photons m^-2^ s^-1^. In addition, the maximum rate of increase in *g*_s_ (d*g*_s_/d*t*_max_) and a lag in time for d*g*_s_/d*t* to reach d*g*_s_/d*t*_max_ (*λ*) were calculated to evaluate the stomatal opening speed during the photosynthetic induction, as described in Sakoda et al. (2021).

### 2.7 Statistical analysis

The significance of variation in each parameter was evaluated between the single leaf and whole plant by one-way analysis of variance (ANOVA) or among the leaves at different ages by repeated measures ANOVA at *p*< 0.05 and 0.01. The correlation among the parameters was analyzed by a using tool in the Seaborn libraries of Python. These analyses were conducted with the individual replicates for each parameter. The significance of correlations among the parameters was evaluated using Pearson’s correlation analysis at *p*< 0.05 and 0.01. Statistical analyses were performed by using R version 4. 3. 3 (R Foundation for Statistical Computing, Vienna, Austria).

## 3 Results

### 3.1 Effects of the fan speed and flow rate on the photosynthetic response to light

**Figure 2:**
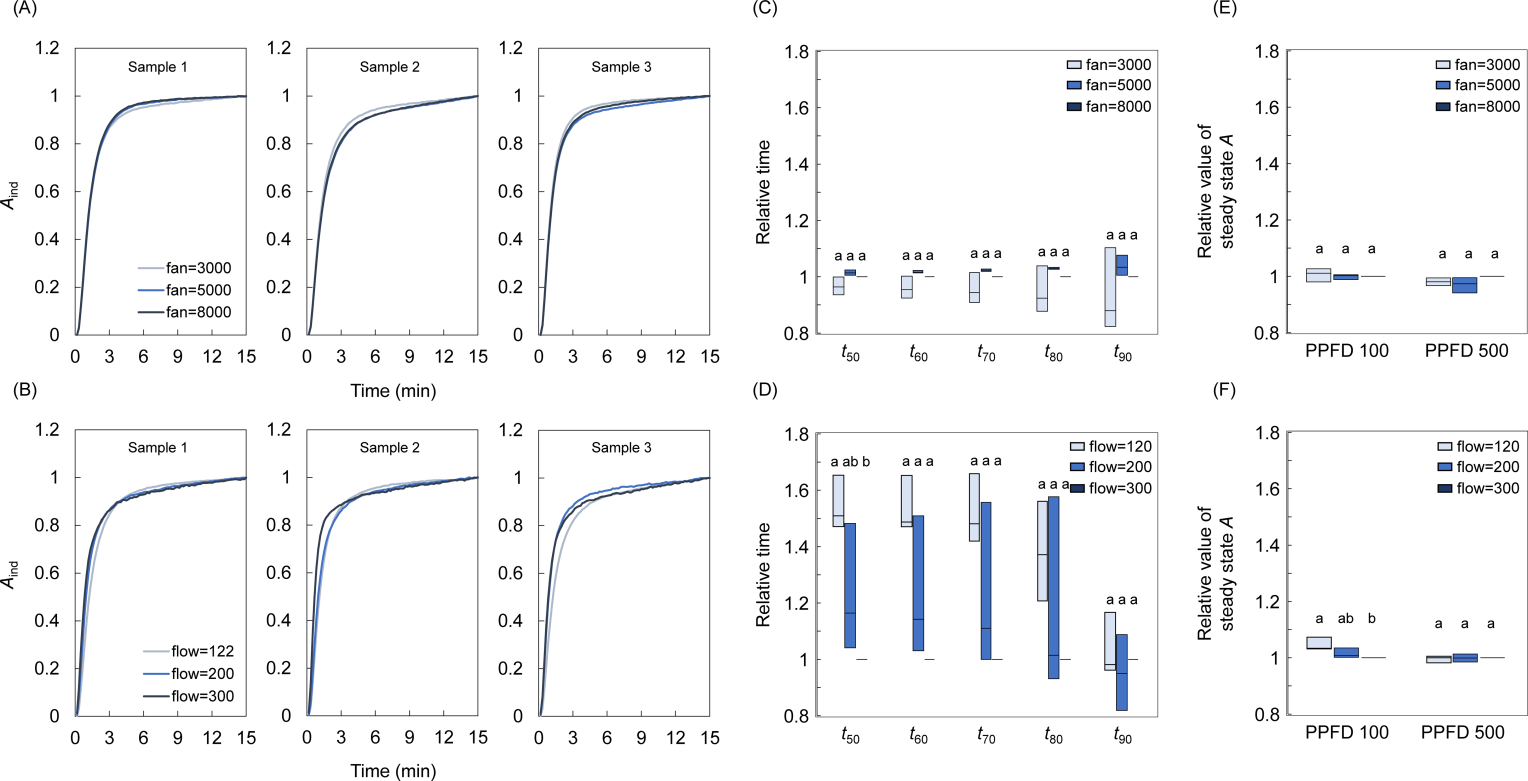
The effect of the fan speed and flow rate on the photosynthetic response to light at the single-leaf level. The change in the CO_2_ assimilation rate (*A*) and steady state *A* was measured after a step increase in light from a PPFD of 100 to 500 µmol photons m^-2^ s^-^ in several **(A)** fan speed and **(B)** flow rate conditions: the fan speeds of 3000, 5000, and 8000 rpm at the flow rate of 300 µmol s^-1^, and the flow rates of 122, 200, and 300 µmol s^-1^ at the fan speed of 8000 rpm. We calculated the relative values of the time for *A* to reach 50-90% to maximum value (*t*_50_ - *t*_90_) and steady state *A* at **(C, E)** the fan speeds of 3000 and 5000 to 8000 rpm, and **(D, F)** the flow rates of 122 and 200 to 300 µmol s^-1^. Each boxplot represents 3 replicates. Different letters indicate significant differences among the fan-speed or the flow-rate conditions at *p<* 0.05.

First, we investigated the effect of the fan speed and flow rate on the evaluation of the photosynthetic response to light under steady and non-steady states. The time course of the *A*_ind_ change and steady state *A* after step increase in light from a PPFD of 100 to 500 µmol m^-2^ s^-1^ was evaluated at three different fan speeds and flow rates using the leaf chamber, and three fan speeds using the WP chamber (**Figures 2A, B**, **Supplementary Figure S3**). As for the single-leaf measurements, *t*_50_ *_A_*- *t*_90_ *_A_* and steady state *A* did not differ significantly among the three fan speeds (**Figures 2C, E**). In contrast, higher flow rates resulted in lower *t*_50_ *_A_*-*t*_80_ *_A_* and slightly lower steady state *A* under a PPFD of 100 µmol m^-2^ s^-1^ (*p*<0.05) (**Figures 2D, F**). As for the whole-plant measurements, *t*_50_ *_A_*- *t*_90_ *_A_* did not differ significantly among the three wind speeds, while higher wind speed resulted in slightly lower steady state *A* under a PPFD of 100 µmol m^-2^ s^-1^ (*p*<0.05) (**Supplementary Figure S7**). These results demonstrate that the flow rate has a major effect on the evaluation of the photosynthesis under non-steady state not but steady state, while the wind speed has a minor effect under both states.

### 3.2 Evaluation of the gas-replacement speed and leak effect on *A* calculation for the leaf and whole-plant chambers

**Figure 3:**
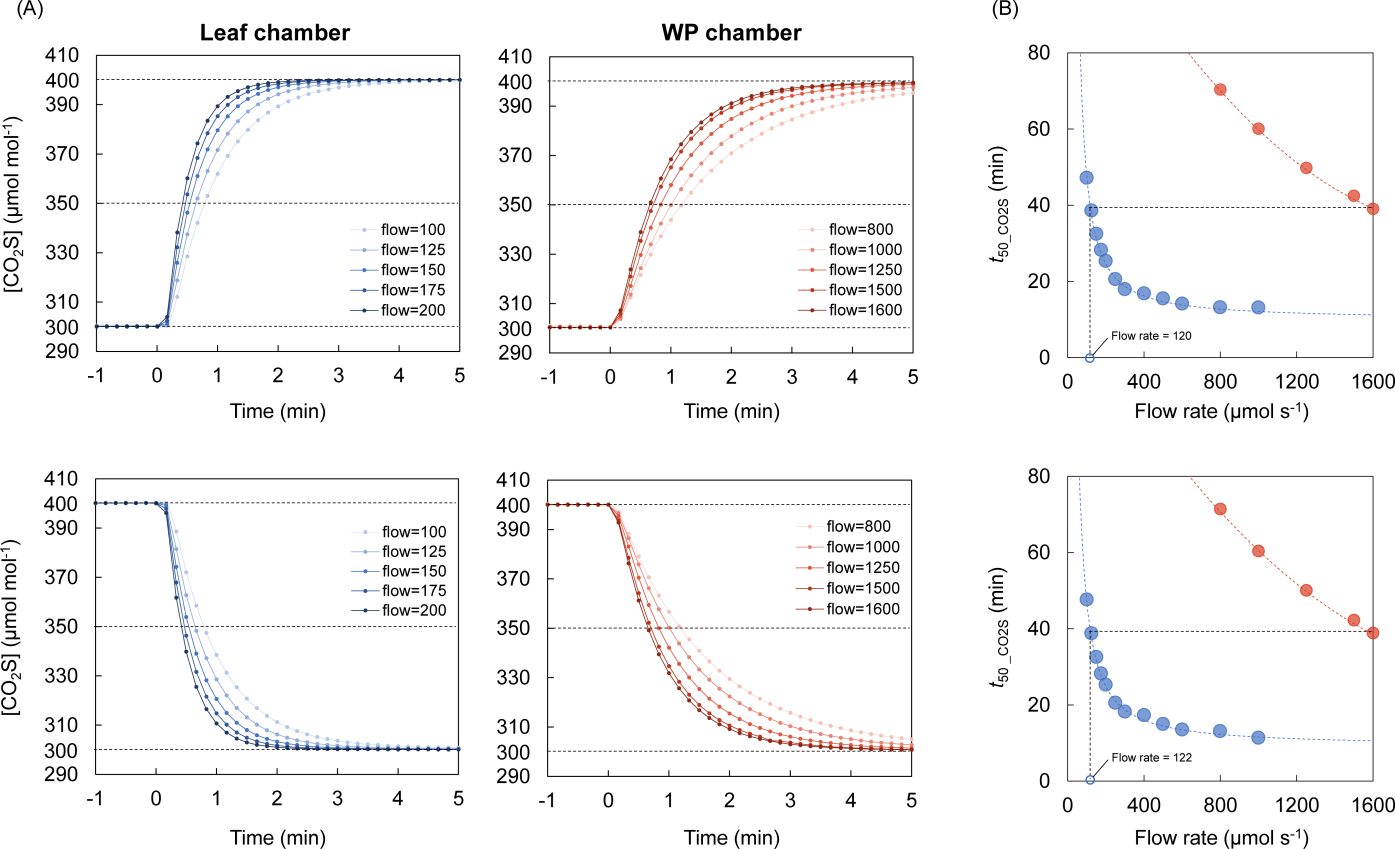
Comparison of gas-replacement speed between the leaf and whole-plant chambers. **(A)** The change in [CO_2_] of sample gas ([CO_2_S]) was compared at the flow rates of 100, 125, 150, 175, and 200 µmol s^-1^ for the leaf chamber and 800, 1000, 1250, 1500, and 1600 µmol s^-1^ for the whole-plant (WP) chamber when [CO_2_] of reference gas ([CO_2_R]) was changed between 300 and 400 µmol mol^-1^. **(B)** The time for [CO_2_S] to reach 50% of the targeted value (*t*_50_CO2S_) is plotted against the flow rates. The functions representing *t*_50_CO2S_ as a variable of the flow rate are shown as the dashed line for the leaf and WP chambers.

We evaluated the time course of the [CO_2_S] changes when the targeted [CO_2_R] was changed between 300 and 400 µmol mol^-1^ or vice versa in both leaf and WP chambers (**Figure 3A**). In both chambers, higher flow rates led to faster gas replacement. The functions representing *t*_50_CO2S_ as a variable of flow rate could be expressed as an exponential function for the leaf chamber and as a quadratic function for the WP chamber (**Figure 3B**). Based on these functions, *t*_50_CO2S_ will be equivalent between the two chambers when the flow rate is 1600 μmol s^-1^ for the WP chamber and 120-122 μmol s^-1^ for the leaf chamber. These flow rate settings are expected to equalize the gas-replacement speed between two chambers and thus were adopted to the gas exchange measurement to compare the photosynthetic response to light between the single leaf and whole plant.

To evaluate the leak effect on calculated *A* for two chambers, we compared the difference in [CO_2_R] and [CO_2_S] (ΔCO_2_) when [CO_2_R] is stable at 300 or 400 μmol mol^-1^ under the flow rate of 125 μmol s^-1^ for the leaf chamber and 1600 μmol s^-1^ for the WP chamber without the plants (**Supplementary Figure S8**). When [CO_2_R] was changed from 300 to 400 μmol mol^-1^, ΔCO_2_ was -0.32 μmol mol^-1^ at [CO_2_R] of 300 μmol mol^-1^ and -0.17 μmol mol^-1^ at [CO_2_R] of 400 μmol mol^-1^ for the leaf chamber and -0.38 μmol mol^-1^ at [CO_2_R] of 300 μmol mol^-1^ and 0.13 μmol mol^-1^ at [CO_2_R] of 400 μmol mol^-1^ for the WP chamber. When [CO_2_R] was changed from 400 to 300 μmol mol^-1^, ΔCO_2_ was -0.31 μmol mol^-1^ at [CO_2_R] of 300 μmol mol^-1^ and -0.27 μmol mol^-1^ at [CO_2_R] of 400 μmol mol^-1^ for the leaf chamber and -0.38 μmol mol^-1^ at [CO_2_R] of 300 μmol mol^-1^ and -0.12 μmol mol^-1^ at [CO_2_R] of 400 μmol mol^-1^ for the WP chamber. Moreover, we calculated the percentage of ΔCO_2_ derived from the leak to ΔCO_2_ during the photosynthetic induction under the singular changing light condition. ΔCO_2_ derived from the leak was assumed to be -0.32 μmol mol^-1^ for the leaf chamber and -0.38μmol mol^-1^ for the WP chamber. The percentage was stable at 18.5% for the leaf chamber and 6.5% for the WP chamber under the initial darkness and subsequently increased and decreased substantially during the photosynthetic induction. Importantly, the percentage was less than 5% at 1.5 min after a step increase in light, confirming the minor leak effect on calculated *A* under steady and non-steady states, except for that under darkness.

### 3.3 Photosynthetic responses to steady and fluctuating light conditions at single-leaf and whole-plant levels

**Figure 4:**
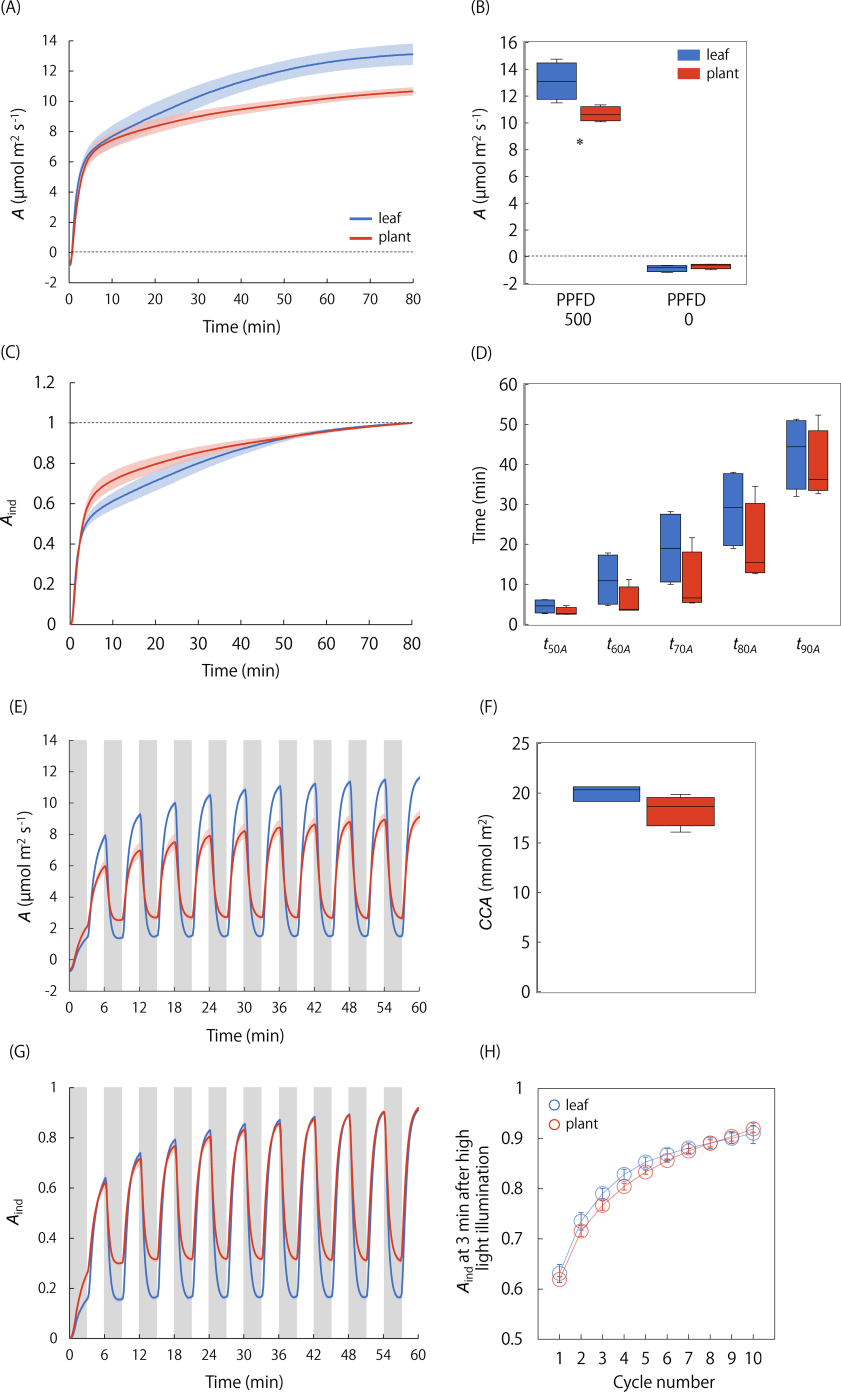
Comparison of photosynthetic response to light between the single leaf and whole plant. **(A)** The changes in *A* and **(B)** steady state *A* were measured after a step increase in light from darkness to a PPFD of 500 µmol photons m^-2^ s^-1^ in the single leaf and whole plant. **(C)** The changes in the induction state of *A* (*A*_ind_) and **(D)** the time for *A*_ind_ to reach 50-90% to maximum value were compared between the single leaf and whole plant. In addition, **(E)** the changes in *A* and **(F)** cumulative CO_2_ assimilation (*CCA*) under a repeated changing light conditions between a PPFD of 60 (gray boxes) and 500 µmol photons m^-2^ s^-1^ were measured. **(G)** The changes in *A*_ind_ and **(H)** *A*_ind_ at 3 min after high-light illumination at each cycle were compared between the single leaf and whole plant. Each boxplot represents 4-6 replicates. * indicates significant differences between the single leaf and whole plant at *p<* 0.05.

**Figure 5:**
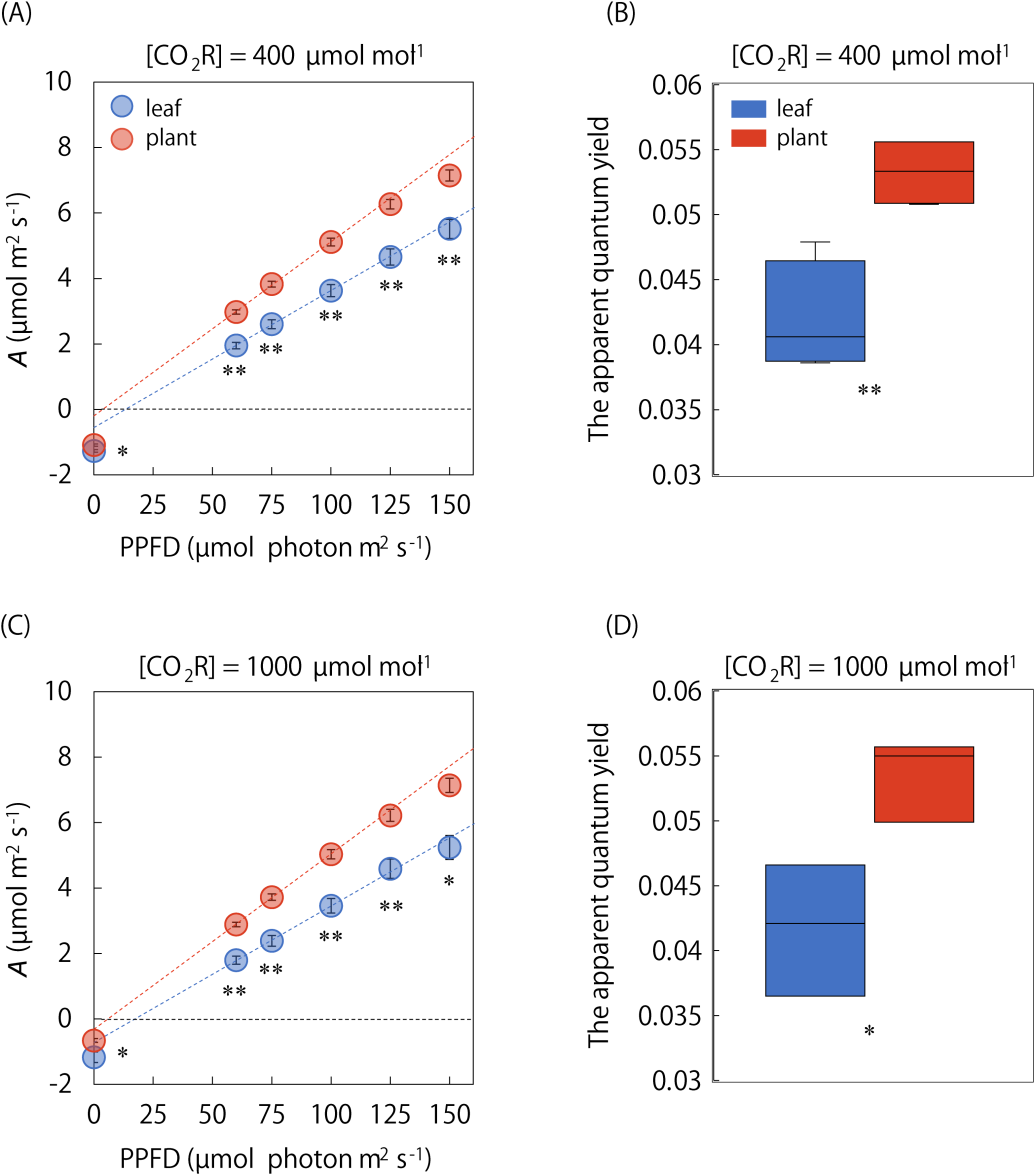
Comparison of *A*-Q curve between the single leaf and whole plant. The light response of steady state *A* was measured at PPFDs of 0, 60, 75, 100, 125, and 150 µmol photons m^-2^ s^-1^, and the apparent quantum yield was compared between the single leaf and whole plant under [CO_2_R] of **(A, B)** 400 and **(C, D)** 1000 µmol mol^-1^. The plots in *A*-Q curve and boxplot represent 3-6 replicates. Vertical bars in panel **(A)** and **(C)** indicate the standard error. * and ** indicate significant differences between the single leaf and whole plant at *p<* 0.05 and 0.01, respectively.

We compared *A* under steady and non-steady states between the single leaf and whole plant under the singular and repeatedly changing light conditions (**Figures 4**, **5**). *A*_max_ was significantly lower in the whole plant than in the single leaf, whereas the difference in *A*_min_ was not significant (**Figure 4B**). On the other hand, the *A*-Q curve analysis showed that steady-state *A* under a PPFD ranging from 0 to 150 μmol·photons·m^-2^·s^-1^ was significantly higher in the whole plant than in the single leaf, and the initial slope value was higher in the whole plant (**Figure 5**). In addition, *A*-Q curves obtained at the single leaf and whole plant level was quite similar under [CO_2_R] of 400 and 1000 µmol mol^-1^. These results indicate that the apparent quantum yield is higher in a whole plant than in a single leaf under low-light conditions, independent of the stomatal limitation.

Under the singular changing light condition, the speed of photosynthetic induction was not significantly different between the single leaf and whole plant (**Figures 4C, D**). The equivalence in the RH is confirmed for two chambers since RH varied from 60.9 to 74.0% in the leaf chamber and from 63.8 to 77.5% in the WP chamber, respectively, during the measurements (**Supplementary Figure S9**). Under the repeatedly changing light condition, *A* under a PPFD of 500 μmol·photons·m^-2^·s^-1^ was higher in the single leaf than in the whole plant, while *A* under a PPFD of 60 μmol·photons·m^-2^·s^-1^ was higher in the whole plant, resulting in no significant difference in *CCA* between the single leaf and whole plant (**Figures 4E, F**). There was no significant difference in the speed of photosynthetic induction after a step increase in light between the single leaf and whole plant as *A*_ind_ at 3 min under a high light was similar in all the cycles (**Figures 4G, H**).

### 3.4 Effect of leaf aging on photosynthetic response to light

**Figure 6:**
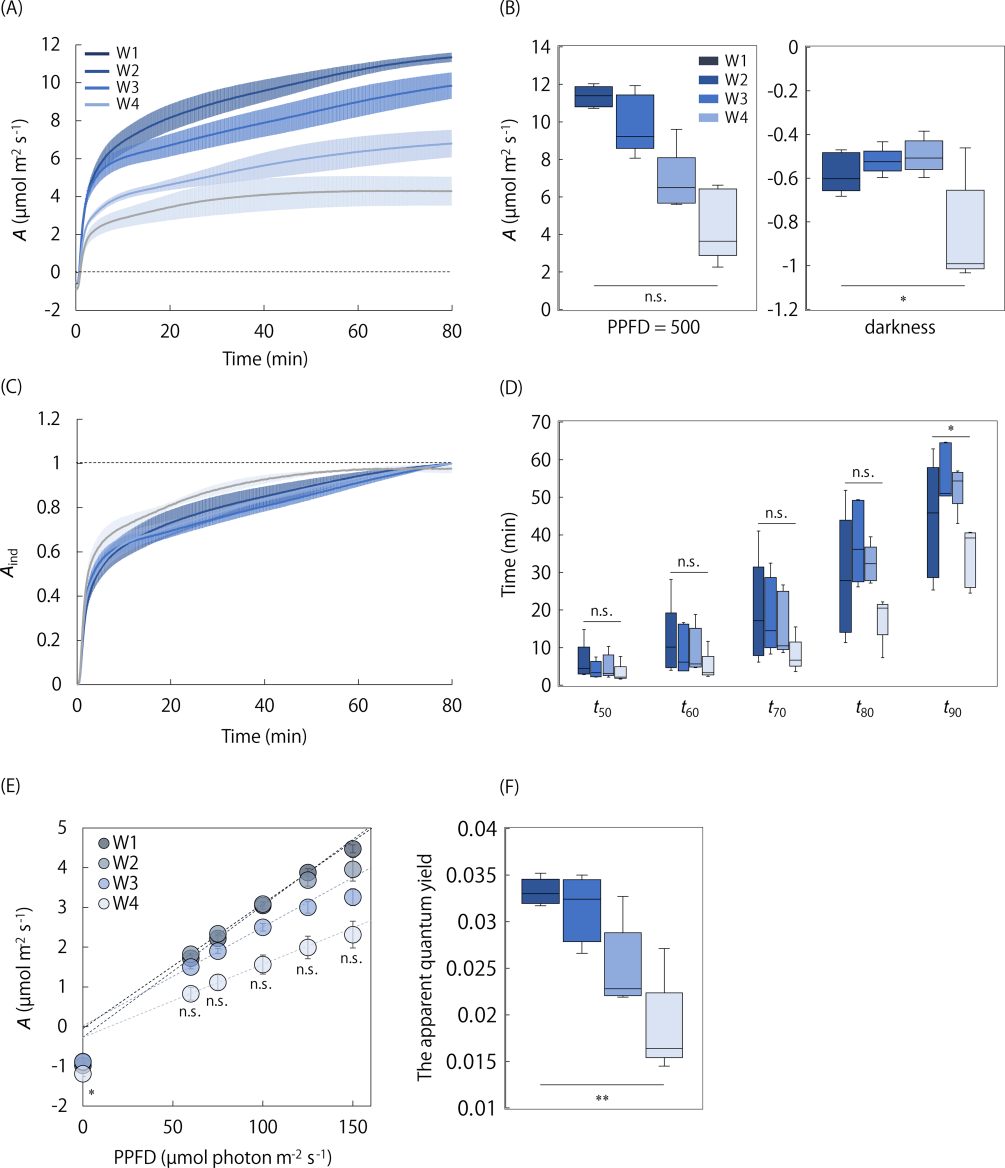
The leaf aging effect on the photosynthetic response to light under steady and non-steady states. **(A)** The changes in *A* and **(B)** steady state *A* were measured after a step increase in light from darkness to a PPFD of 500 µmol photons m^-2^ s^-1^ in the single leaf at the four aging stages (W1-W4). **(C)** The changes in *A*_ind_ and **(D)** the time for *A*_ind_ to reach 50-90% to maximum value were evaluated. The light response of steady state *A* and the apparent quantum yield were evaluated at the four stages, as described in **Figure 4**. The plots in *A*-Q curve and boxplot represent 5-6 replicates. Vertical bars in panel **(A, C, E)** indicate the standard error. The n.s. indicates no significant difference among the four stages. * and ** indicate significant differences among the four stages at *p<* 0.05 and 0.01, respectively.

**Figure 7:**
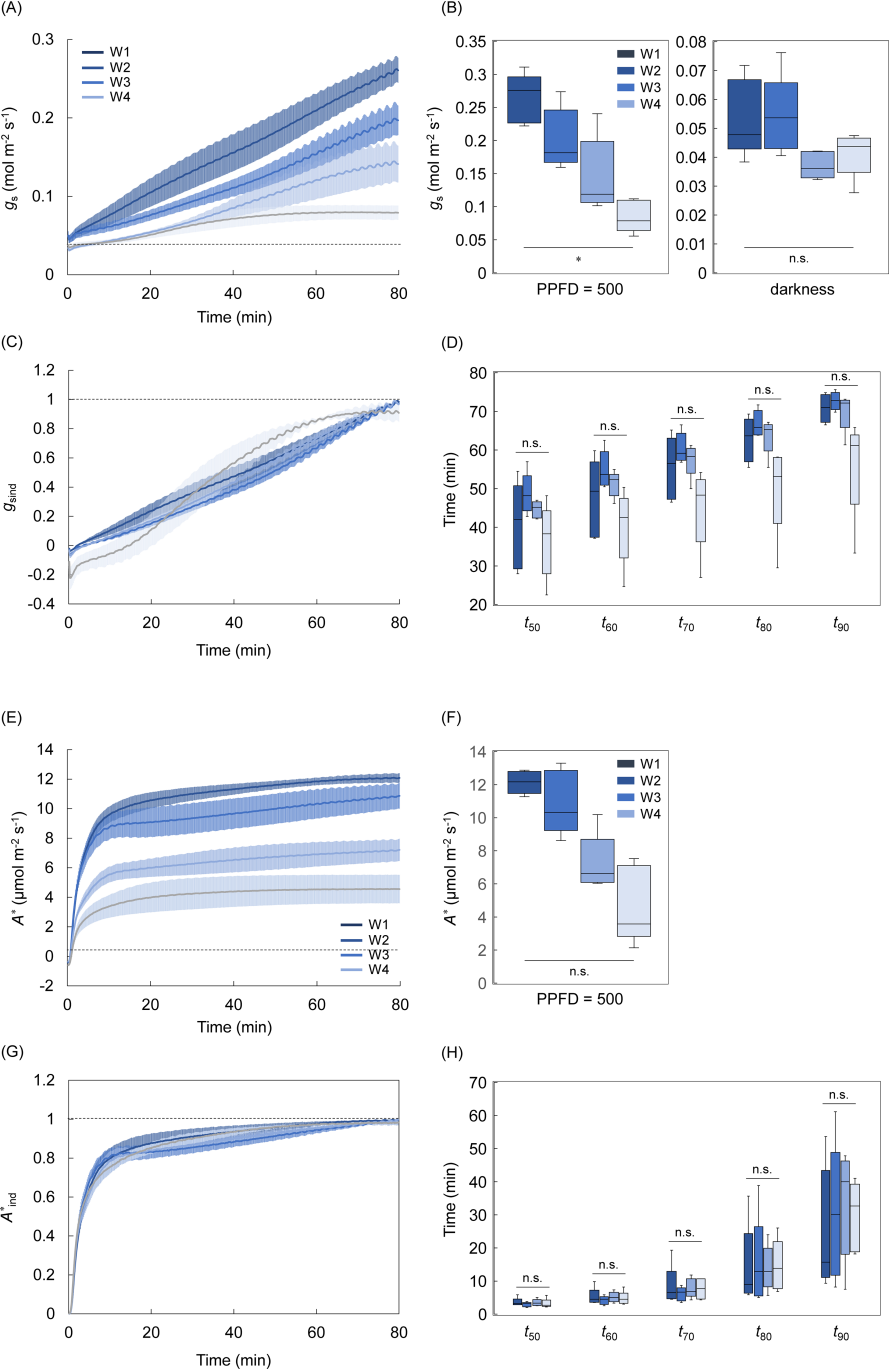
The leaf aging effect on the light response of stomatal and non-stomatal limitations under steady and non-steady states. **(A, B)** The changes in a stomatal conductance (*g*_s_) and **(E, F)** *A* corrected for stomatal limitation (A^*^) and their steady-state values were measured after a step increase in light from darkness to a PPFD of 500 µmol photons m^-2^ s^-1^ in the single leaf at the four aging stages (W1-W4). **(C, G)** The change in the induction state of *g*_s_ (*g*_sind_) and A^*^ (*A* ^*ind^), and **(D, H)** the time for *g*_sind_ and A^*^ ^ind^ to reach 50-90% to maximum value was evaluated. The boxplot represents 5-6 replicates. Vertical bars in panels **(A, C, E, G)** indicate the standard error. The n.s. indicates no significant difference among the four stages. * indicates significant differences among the four stages at *p<* 0.05.

**Figure 8:**
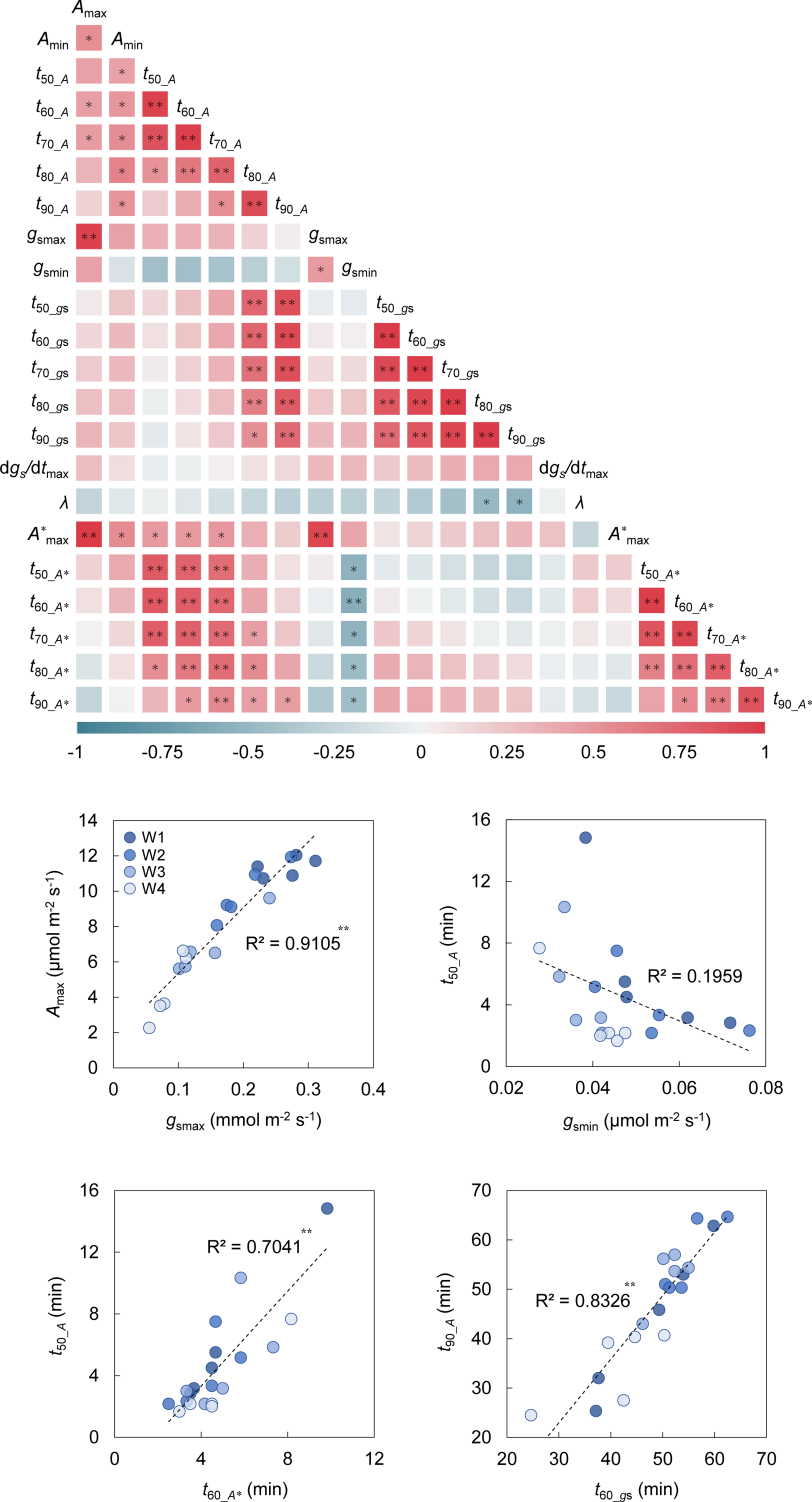
Heatmap of correlation co-efficient among the parameters related to photosynthetic response to light through leaf aging. The Pearson’s co-efficient was evaluated among the parameters related to the photosynthetic response to light under steady and non-steady states. The parameters were obtained in the single leaf at the four aging stages (W1-W4). The parameters are as follows: *A*_max_ and *A*_min_ are maximum and steady-state values of *A* under a PPFD of 500 µmol m^-2^ s^-1^ and darkness, respectively; *t*_50__ *_A_*, *t*_60__ *_A_*, *t*_70__ *_A_*, *t*_80__ *_A_*, *t*_90__ *_A_* are the time for *A* to reach 50-90% to maximum value during induction; *g*_smax_ and *g*_smin_ are maximum and steady-state values of *g*_s_ under a PPFD of 500 µmol m^-2^ s^-1^ and darkness, respectively; *t*_50__ *_g_*_s_, *t*_60__ *_g_*_s_, *t*_70__ *_g_*_s_, *t*_80__ *_g_*_s_, *t*_90__ *_g_*_s_ are the time for *g*_s_ to reach 50-90% to maximum value during induction; d*g*_s_/d*t*_max_ is maximum value of the rate of increase in *g*_s_ (d*g*_s_/d*t*) and a lag in time for d*g*_s_/d*t* to reach d*g*_s_/d*t*_max_ (*λ*) during induction; *A* ^*max^ is maximum value of *A* ^*^ under a PPFD of 500 µmol m^-2^ s^-1^; *t*_50__ _A__*_, *t*_60__ _A__*_, *t*_70__ _A__*_, *t*_80__ _A__*_, *t*_90__ _A__*_ are the time for *A* ^*^ to reach 50-90% to maximum value during induction. * and ** indicate significant correlation between the parameters at *p<* 0.05 and 0.01, respectively.

We investigated the effect of leaf aging on the photosynthetic response to light (**Figures 6**–**8**). Under the singular changing light condition, *A*_max_ reduced through leaf aging, although the variation among the four stages was not significant (**Figures 6A, B**). *A*_min_ changed little, while it showed the significant variation among the four stages. There was no significant variation in *t*_50_ *_A_*- *t*_80_ *_A_* among the four stages (**Figures 6C, D**), but there was significant variation at W4. The *A*-Q curve analysis showed *A* reduced under a PPFD ranging from 60 to 150 µmol m^-2^ s^-1^ (not significant) and the initial slope significantly reduced through leaf aging (**Figures 6E, F**).

*g*_smax_ significantly reduced through leaf aging, while *g*_smin_ changed little (**Figures 7A, B**). Similar to *A*, *A* ^*^ reduced through leaf aging but the variation was not significant among the four stages. There was no significant variation in the speed of *g*_s_ induction, and that of *A* ^*^ induction among all four stages (**Figures 7C, D, G, H**). The *g*_s_ induction tended to be faster at W4 than at the other stages, but this could be considered as an apparent rapid response of *g*_s_, likely due to the relatively small increase in *g*_s_. Although the d*g*_s_/d*t*_max_ and λ was not significantly different among the four stages, the d*g*_s_/d*t*_max_ and λ tended to be higher at W1 and W4, respectively, than at the other stages (**Supplementary Figure S10**).

We conducted a correlation analysis among the parameters related to the photosynthetic response to light under the process of leaf aging. There were significantly positive or negative correlations between the tested parameters (**Figure 8**). The strong correlation between *A*_max_ and *g*_smax_ (*R* = 0.954) suggests that the *A*_max_ reduction through leaf aging can be largely attributed to the degraded performance of CO_2_ diffusion via stomata. *t*_50__ *_A_*, *t*_60__ *_A_*, and *t*_70__ *_A_* strongly correlated with *t*_50__ _A*_, *t*_60__ _A*_, and *t*_70__ _A*_, while *t*_80__ *_A_* and *t*_90__ *_A_* strongly correlated with *t*_50__ *_g_*_s_ *t*_90__ *_g_*_s_. Although *g*_smin_ did not significantly correlate with *t*_50__ *_A_t*_90__ *_A_*, it did with *t*_50__ _A*_t_90__ _A*_.

## 4 Discussion

### 4.1 Novelty of the whole-plant gas exchange measurement system

The biomass production of plants depends on the sum of CO_2_ assimilation for all the photosynthetic apparatuses (Long et al., 2006). This fact has driven previous researchers to develop (1) closed, (2) semi-closed, and (3) open systems for gas exchange measurement with the whole plant (Takahashi et al., 2008). The closed system (Katsura et al., 2006) is suitable for the measurements with large plants, but it is not applicable to a sequential measurement for evaluating the photosynthetic response to short-term environmental changes. Several studies proposed open systems to evaluate the photosynthetic response (van Iersel and Bugbee, 2000; Kölling et al., 2015; Jauregui et al., 2018), but these cannot provide enough time-resolution to capture the photosynthetic induction that typically occurs in a time scale from seconds to hours. The open system employing the small chamber can realize the measurements with the high time-resolution (Tocquin and Périlleux, 2004; Brazel et al., 2024), while it encloses the small plants such as Arabidopsis seedlings and does not enable the photosynthetic response to be compared between the single leaf and whole plant in the same plant. When constructing an open system using larger chamber for larger plants, challenges can arise associated with the environmental regulation (e.g. air temperature and humidity, flow rates, light intensity, [CO2]…) inside the chamber. The larger chamber should make more difficult to homogenize environmental conditions spatiotemporally. In the present study, we designed the WP chamber to be connectable to LI-6800 and to be installable in the growth chamber, enabling the highly precise control of environmental conditions. The developed open system can be applied for gas exchange measurement with the whole plant of Arabidopsis with a diameter ≦ 17 cm, which enables the photosynthetic response to be directly compared between the single leaf and whole plant (**Figures 1**-**4**). It has the potential to extend our understanding of the photosynthetic response to short-term environmental changes from the single-leaf level to the whole-plant level.

### 4.2 Difference in the photosynthetic response to light under steady and non-steady states between the single leaf and whole plant

The photosynthetic response to light under steady and non-steady states has been extensively investigated at the single-leaf level in various plant species (Lawson et al., 2012; Sakoda et al., 2022). However, little is known about the photosynthetic response at the whole-plant level. Land plants typically consist of many leaves differing in terms of spatial position and age. Different photosynthetic responses to the fluctuating light were reported between leaves at different ages (Fukayama and Uchida, 1998) and positions (Acevedo-Siaca et al., 2021) in the same plant. These differences possibly lead to the difference in the observable photosynthetic response between the single leaf and whole plant. Importantly, the present study is the first to reveal the whole-plant photosynthetic response to light under steady and non-steady states and its difference from the single-leaf response. The photosynthetic response to the fluctuating light does not significantly differ between the single leaf and whole plant (**Figure 4**). Arabidopsis forms rosette leaves so that the light environment experienced by each leaf is similar. In addition, the photosynthetic induction was not significantly affected by leaf aging (**Figure 6**). Therefore, it is possible that there is no large difference in the photosynthetic response to fluctuating light among the leaves at different positions and ages, which results in the similar response between the single leaf and whole plant in Arabidopsis. Note that this consideration does not necessarily apply to the plant species that exhibit vertical stem elongation and leaf expansion.

It was previously shown in soybeans that steady state *A* under the saturated light conditions was higher in the whole plant than in the single leaf but that under the sub-saturated light conditions was lower (Wheeler et al., 2003). On the other hand, the present study found that steady state *A* under the low-light conditions and, consequently, the apparent quantum yield was higher in the whole plant than in the single leaf of Arabidopsis (**Figure 5**). The apparent quantum yield is largely determined by the efficiency to absorb the light by photosynthetic pigments (Gabrielsen, 1948; Leverenz, 1987), and to utilize the absorbed light for CO_2_ fixation (Skillman, 2008). The efficiency to utilize the absorbed light is affected by the photorespiration and non-photochemical quenching (NPQ), which consumes the light energy in ways that do not contribute to photosynthesis. Considering these facts, the higher apparent quantum yield in the whole plant than the single leaf is hypothesized to be attributed to the following aspects: the higher light absorption efficiency due to the higher chlorophyll content, and/or the higher light utilization efficiency due to lower energy consumption by photorespiration and NPQ, in young leaves than old leaves in Arabidopsis. This hypothesis is partly supported by the fact that the apparent quantum yield linearly reduced through leaf aging in Arabidopsis (**Figure 6**). In addition, young leaves showed a lower photorespiration level than old leaves in tobacco [*Nicotiana tabacum* (L.)] and several species of genus *citrus* (Salin and Homann, 1971), and the lower NPQ level in Arabidopsis (Bielczynski et al., 2017) and barley [*Hordeum vulgare* (L.)] (Shimakawa et al., 2022).

In the present study, it is necessary to acknowledge the limitations in the comparison of single leaf and whole plant measurements, which are associated with (1) leaf temperature, (2) mutual leaf shading, and (3) boundary layer conductance. Both single leaf and whole plant measurements were conducted under the similar air temperature and light conditions, leading to the expectation that leaf temperatures would be similar between both the measurements. ALA/PLA was higher at the later growth stage compared to the early growth stage (**Supplementary Figure S5**), indicating that the degree of mutual leaf shading was greater in the later growth stage. As growth progressed, mutual leaf shading might cause the light intensity on some leaves to be lower than on other positions, potentially affecting the *A* evaluation in the whole plant measurements. Indeed, there was no significant change in *A* for the whole plant throughout the growth period. It suggests that mutual leaf shading would have a minor impact on the *A* evaluation with the whole plant. In addition, the differences in wind speed resulted in a slight change in the photosynthetic response to light under steady and non-steady states in the single-leaf and whole-plant measurements, respectively (**Figure 2**, **Supplementary Figure S7**). This result suggests that the differences in boundary layer conductance would not be a major factor underlying the differences in the photosynthetic response to light between a single leaf and whole plant. In addition, the difference in the light conditions for the plant cultivation (a PPFD of 100 µmol m^-2^ s^-1^) and gas exchange measurement (a PPFD of 60 to 500 µmol m^-2^ s^-1^) can affect the evaluation of photosynthetic characteristics in the present study, although often overlooked in other previous studies. Future research is desirable to test this potential effect on photosynthesis in plants.

### 4.3 Leaf aging effect on photosynthetic response to light under steady and non-steady states

The leaf aging effect has been investigated on photosynthesis under steady and non-steady states. Leaf aging decreases steady state *A*, accompanied by the reduction in *g*_s_, and the activity of biochemical processes related to RuBP carboxylation and regeneration in plants (Makino et al., 1984; Vos and Oyarzún, 1987; Jiang et al., 1993; Loreto et al., 1994), as shown in the present study (**Figures 6**-**8**). It was previously shown that leaf aging delayed the photosynthetic induction owing to the increased stomatal and non-stomatal limitations in tomatoes (Zhang et al., 2022). On the other hand, the faster photosynthetic induction was reported in an older leaf than in an younger leaf, associated with the lower ratio of ribulose-1,5-bisphospate carboxylase/oxygenase (Rubisco) to Rubisco activase contents in rice (Fukayama and Uchida, 1998). In Arabidopsis, leaf aging had little impact on the speed of *A* induction as well as *g*_s_ and *A* ^*^ inductions (**Figures 6**, **7**). These results suggest that the leaf aging effect on the photosynthetic response under non-steady state would differ among the plant species, depending on the changing pattern of the stomatal and non-stomatal limitations during the aging process.

The present study partly confirms that the photosynthetic response to light in the whole plant depends on the temporal variation in the photosynthetic response of the single leaf, which might be applicable to other plant species. In order to test for this hypothesis, it is required (1) to develop the open system which enables the whole-plant measurement with larger plants, and (2) to evaluate the temporal variation in the single-leaf photosynthetic response in several plant species. In future, further research is expected to tackle these challenges.

## Data Availability

The raw data supporting the conclusions of this article will be made available by the authors, without undue reservation.
