# Supplementary material for: Difference in single-leaf and whole-plant photosynthetic response to light under steady and non-steady states in Arabidopsis thaliana
Source: Front Plant Sci. 2025 Feb 18;16:1532522. doi: 10.3389/fpls.2025.1532522 (PMC11876398; doi:10.3389/fpls.2025.1532522)
Supplement: Supplementary file 1 [file DataSheet1.pdf]

Supplementary Material

1.1 Supplementary Figures

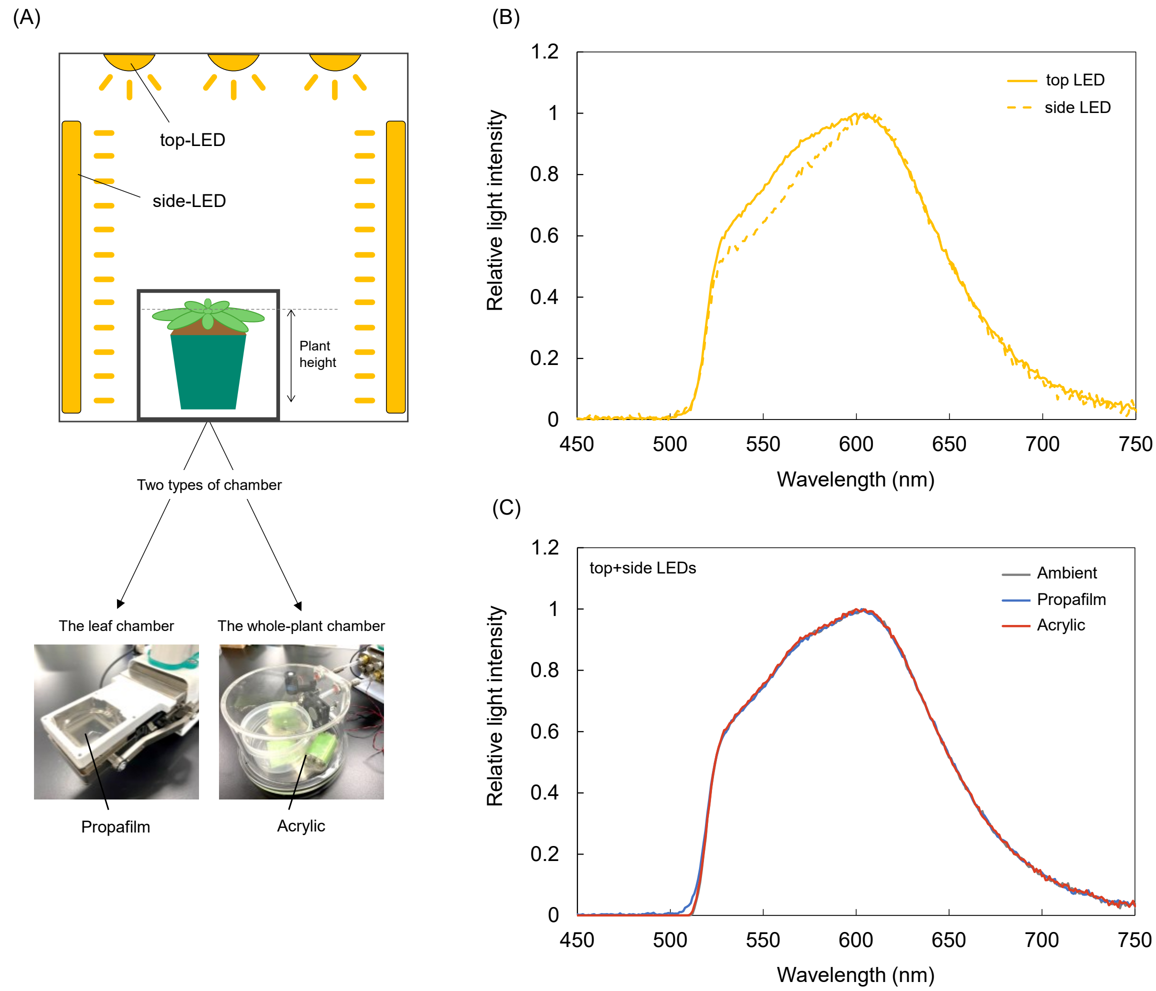

Figure S1. Schematic overview of the growth chamber.

(A) Overview of the growth chamber where gas exchange measurements were conducted. (B) The growth chamber was equipped with LEDs at the top and side positions with different spectrums. (C) The leaf and whole-plant chambers were located at the bottom position of the growth chamber, and spectrums of the transmitted light through both chambers was found to be similar.

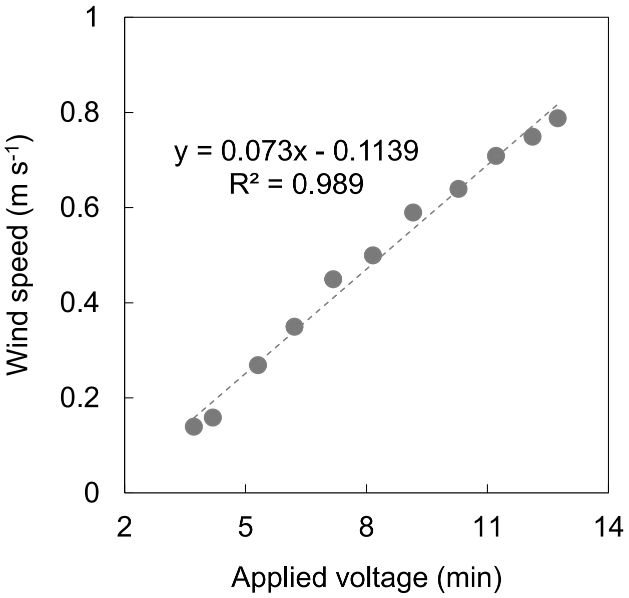

Figure S2. Relationship between the applied voltage to air-mixing fan and wind speed in the whole plant chamber.

The relationship between the applied voltage to air-mixing fan and wind speed in the whole plant chamber fits the linear regression.

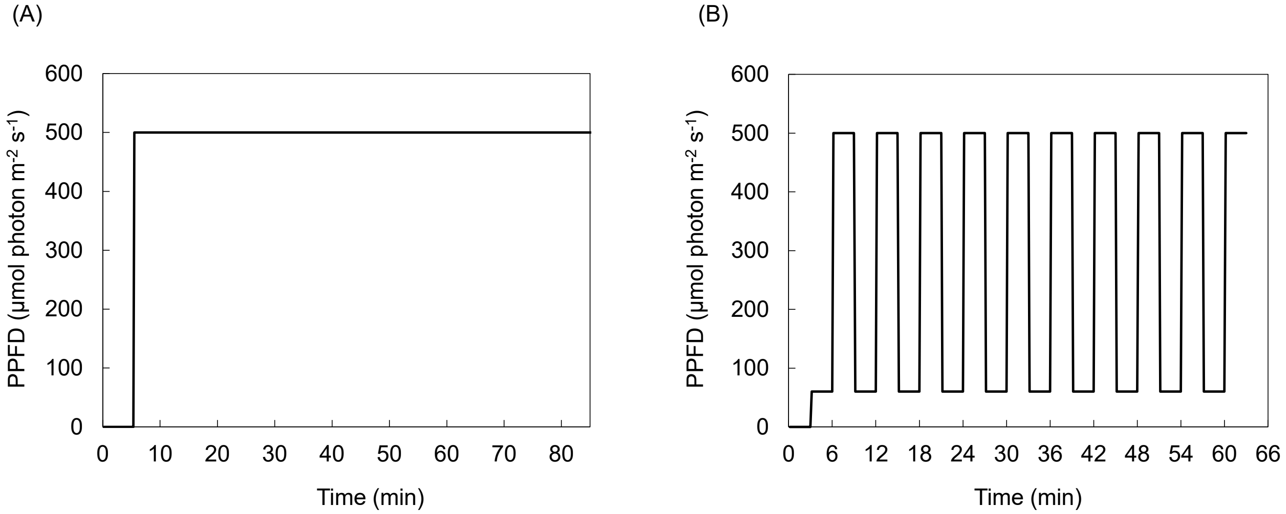

Figure S3. Singular and repeatedly changing light conditions.

Gas exchange measurements were conducted under (A) a singular changing light condition comprising darkness for 5 min and a PPFD of 500 μmol photons m^{-2} s^{-1} for 80 min and (B) a repeatedly changing light condition comprising darkness for 5 min in the beginning, followed by 10 cycles of a PPFD of 60 and 500 μmol·photons·m^{-2}·s^{-1} for 3 min, respectively.

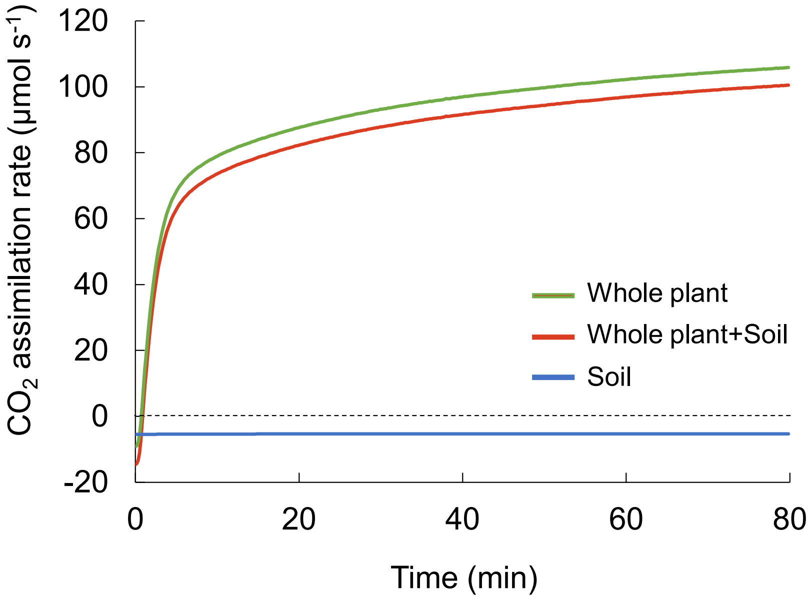

Figure S4. Example of the change in CO_2 assimilation rate after a step increase in light by the whole plant and soil.

A representative example is shown for the change in CO_2 assimilation rate derived from the whole plant and soil after a step increase in light from darkness to a PPFD of 500 µmol photons m^{-2} s^{-1}.

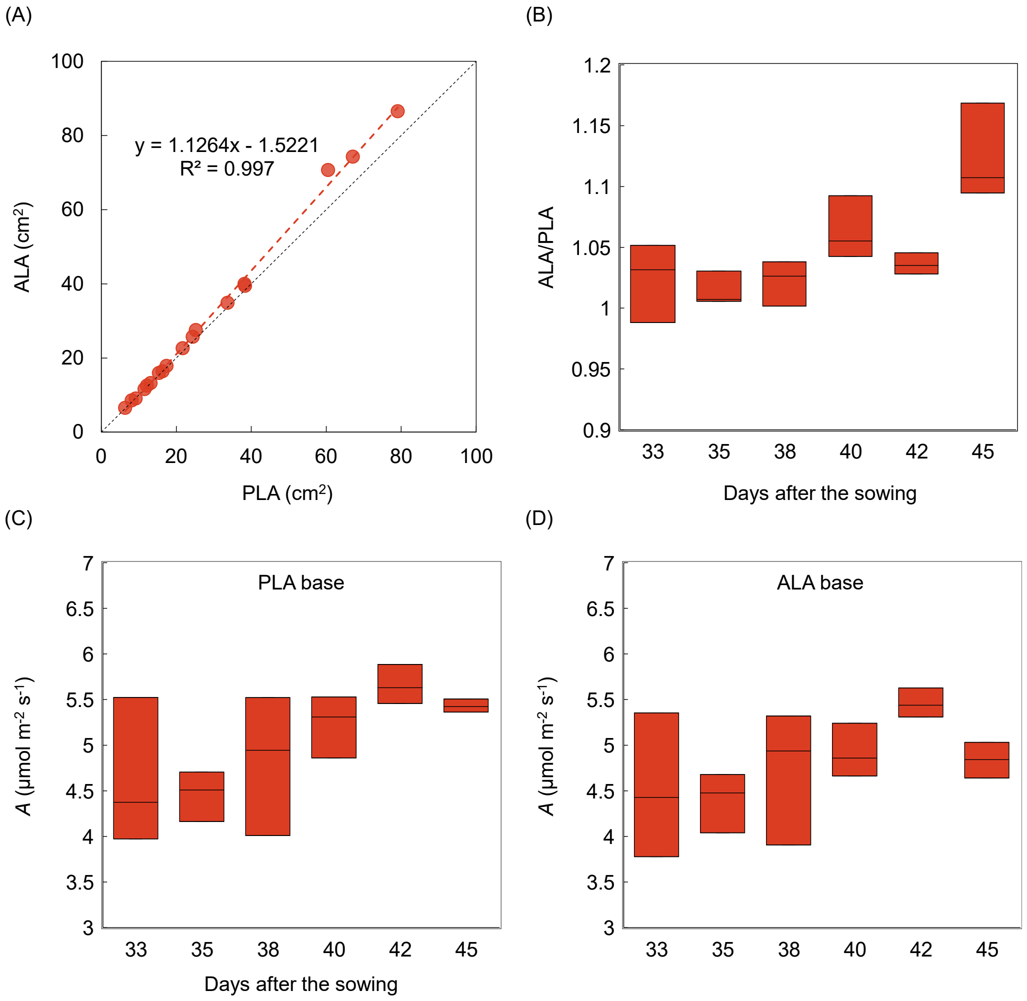

Figure S5. Effects of the leaf area estimation on the calculated *A* for the whole plant.

(A, B) The relationship between the projected leaf area (PLA), and actual leaf area (ALA) measured by the destruction of plant to separate each leaf fits the linear regression. The CO_2 assimilation rate per unit leaf area (*A*) was calculated on the basis of the (C) PLA and (D) ALA. Box plots represent 3 replicates.

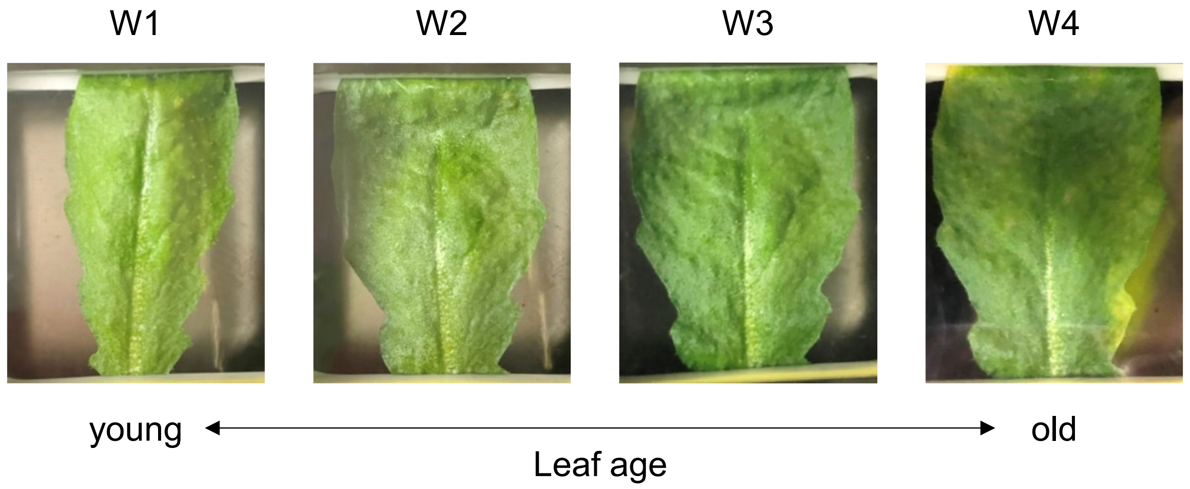

Figure S6. Example of an appearance of the leaves used for the leaf-aging effect analysis.

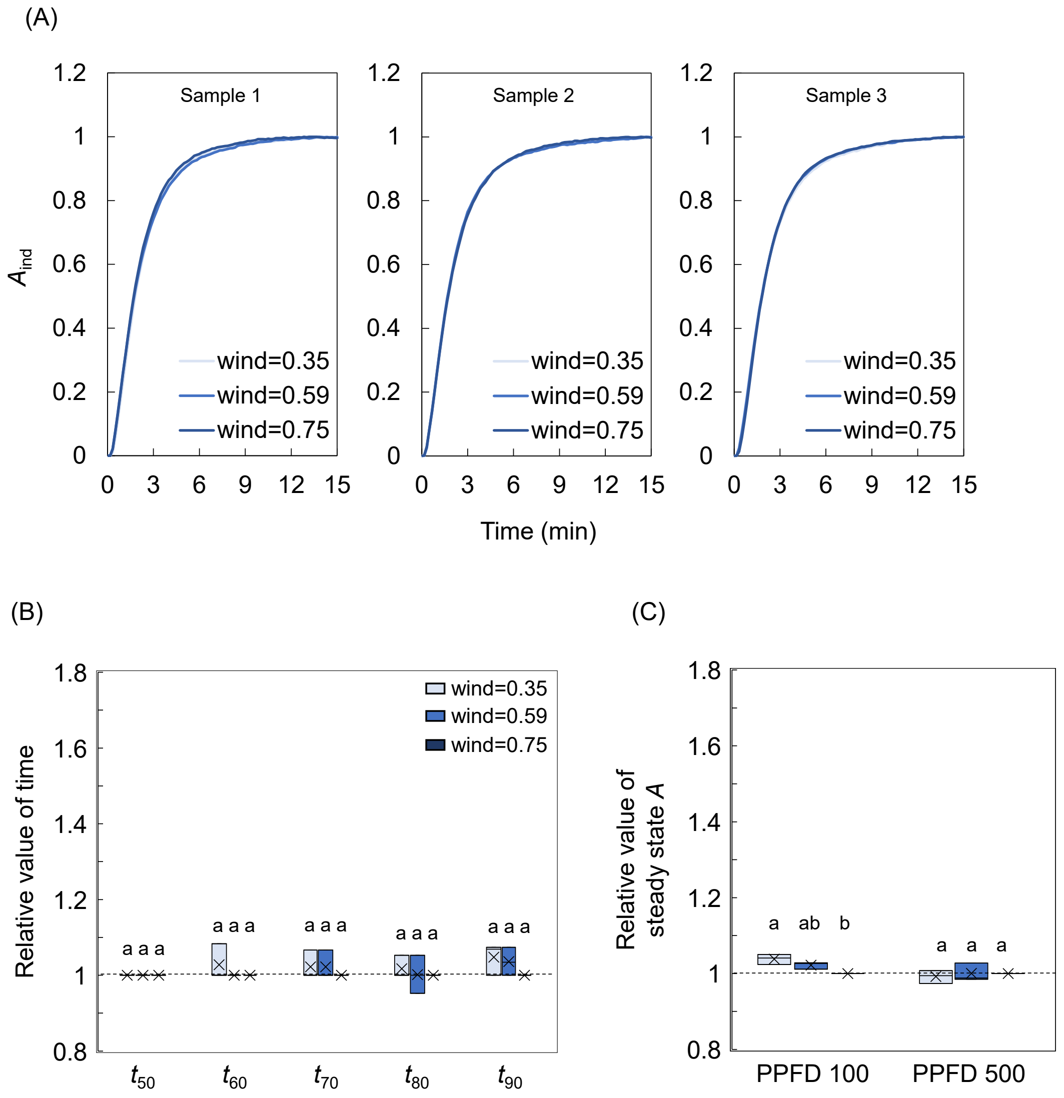

Figure S7. The effect of the wind speed on the photosynthetic response to light at the whole-plant level.

(A) The change in the CO_2 assimilation rate (A) and steady state A was measured after a step increase in light from a PPFD of 100 to 500 µmol photons m^{-2} s^{-} at wind speeds of 0.35, 0.59, or 0.75 m s^{-1} with a flow rate of 1600 µmol s^{-1}. We calculated the relative values of (B) the time for A to reach 50-90% to maximum value and (C) steady state A at each wind speed. Different letters indicate significant differences among the fan-speed or the flow-rate conditions at $p < 0.05$ (n = 3).

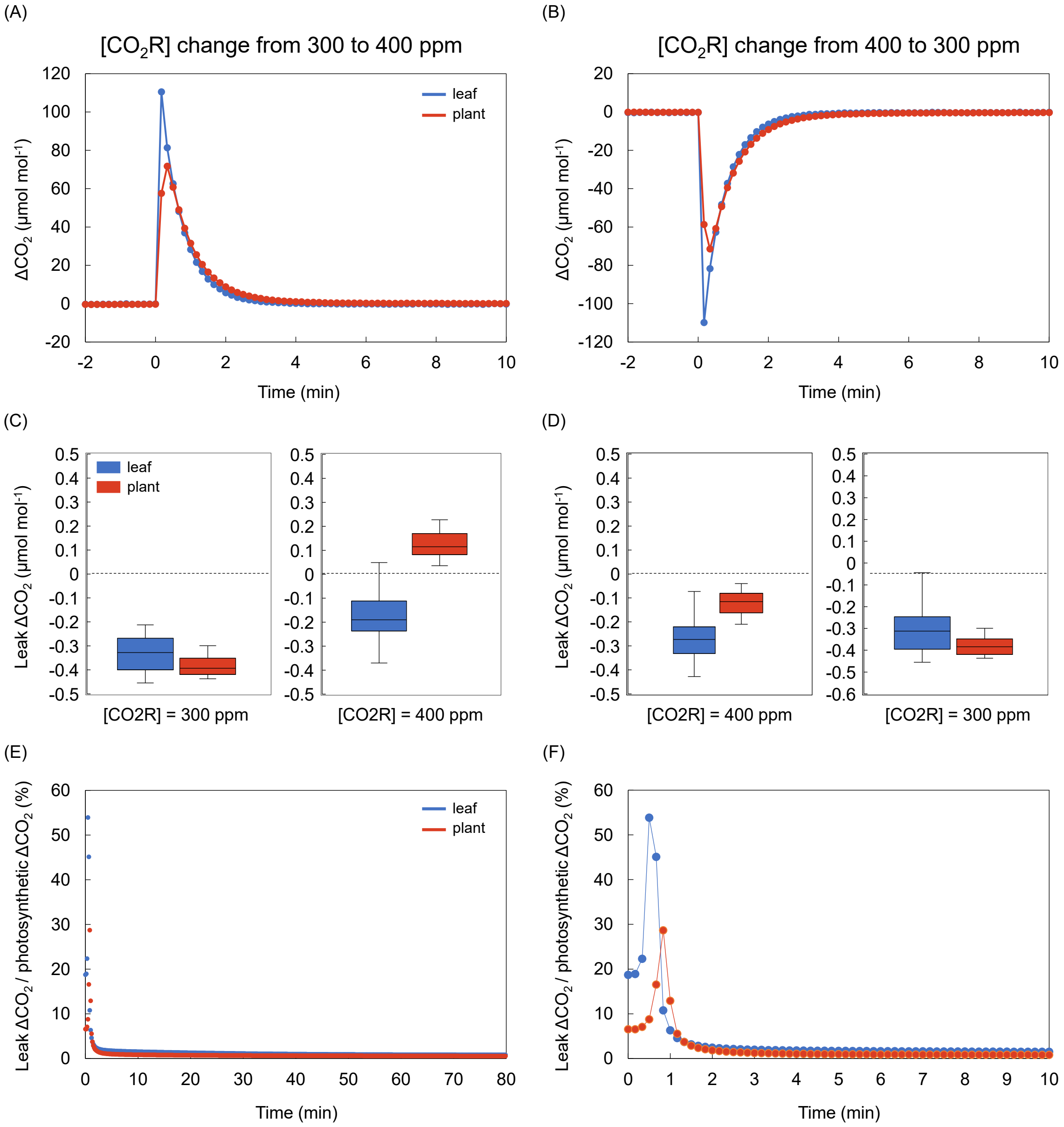

Figure S8. Gas-leak effect on the detection of the difference in the CO_2 concentration between reference and sample gases in the leaf and whole plant chambers.

The difference in [CO_2] between reference and sample gases (ΔCO_2) was evaluated when targeted [CO_2] of reference gas ([CO_2R]) is changed from (A) 300 to 400 μmol mol^{-1} or (B) 400 to 300 μmol mol^{-1}. (C,D) The ΔCO_2 derived from the gas leak between the inside and outside chamber (leak ΔCO_2) was estimated as the steady state ΔCO_2 at 300 and 400 μmol mol^{-1}. (E,F) The percentage of leak ΔCO_2 to ΔCO_2 derived from photosynthesis (photosynthetic ΔCO_2) was calculated after a step increase in light from darkness to a PPFD of 500 μmol photons m^{-2} s^{-1}. Box plots represent 17-18 replicates.

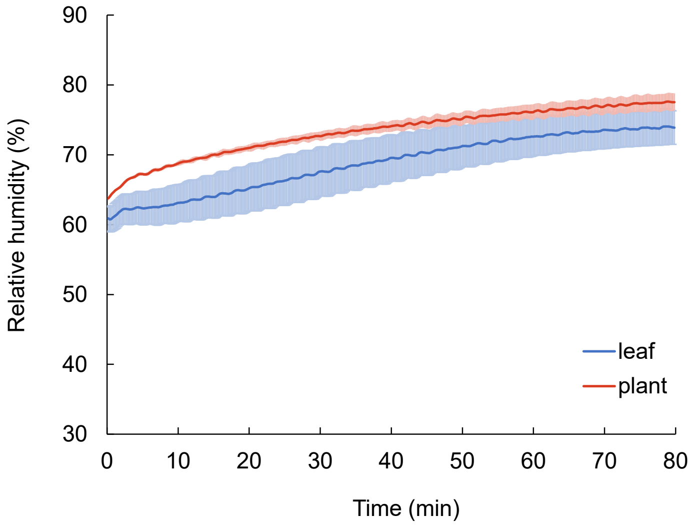

Figure S9. Comparison of the change in the relative humidity after a step increase in light between the single leaf and whole plant.

(A) The change in the relative humidity was measured after a step increase in light from darkness to a PPFD of 500 µmol photons m^{-2} s^{-1} in the single leaf and whole plant. (n=4-6)

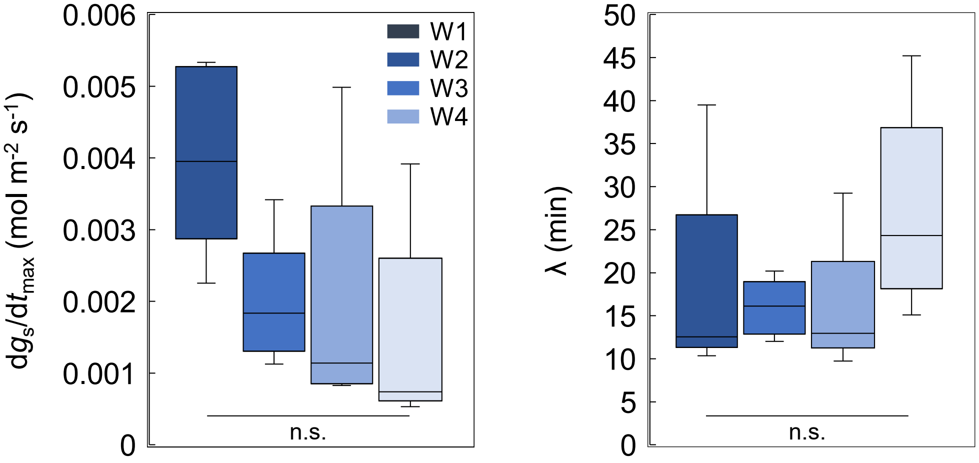

Figure S10. The leaf aging effect on the response of stomatal opening to light under steady and non-steady states.

The maximum rate of increase in the stomatal conductance (g_s) (dg_s/dt_{max}) and a lag in time for dg_s/dt to reach dg_s/dt_{max} (λ) were measured after a step increase in light from darkness to a PPFD of 500 µmol photons m^{-2} s^{-1} in the single leaf at the four aging stages (W1-W4). The boxplot represents 5-6 replicates. The n.s. indicates no significant variation among the four stages.
